# Influence of Shape on the Haptic Size Aftereffect

**DOI:** 10.1371/journal.pone.0088729

**Published:** 2014-02-19

**Authors:** Astrid M. L. Kappers, Wouter M. Bergmann Tiest

**Affiliations:** MOVE Research Institute, Faculty of Human Movement Sciences, VU University Amsterdam, Amsterdam, The Netherlands; University of Ottawa, Canada

## Abstract

Recently, we showed a strong haptic size aftereffect by means of a size bisection task: after adaptation to a large sphere, subsequently grasped smaller test spheres felt even smaller, and vice versa. In the current study, we questioned whether the strength of this aftereffect depends on shape. In four experimental conditions, we determined the aftereffect after adaptation to spheres and tetrahedra and subsequent testing also with spheres and tetrahedra. The results showed a clear influence of shape: the haptic aftereffect was much stronger if adaptation and test stimuli were identical in shape than if their shapes were different. Therefore, it would be more appropriate to term such aftereffects haptic shape-size aftereffects, as size alone could not be the determining factor. This influence of shape suggests that higher cortical areas are involved in this aftereffect and that it cannot be due to adaptation of peripheral receptors. An additional finding is that the geometric property or combination of properties participants use in the haptic size bisection task varies widely over participants, although participants themselves are quite consistent.

## Introduction

Aftereffects, changes in perception due to a previous stimulus, occur in all modalities and the study of these provides insights into the processing of information (e.g. [1]). Probably one of the first mentions of an aftereffect in touch was made by Locke, a seventeenth century philosopher [2]. He described an experiment in which participants had to put one hand in a bowl of hot water and the other in cold water. When, after some time, both hands were placed in a bowl of tepid water, the water felt cold to the hand previously exposed to the hot water, and hot to the hand used to the cold water. So although both hands were exposed to water of the same temperature, the perception depended strongly on the previous stimulation: a temperature aftereffect.

In the current study, we were interested in haptic size aftereffects, particularly how these depend on the shapes of both the adaptation stimulus (the stimulus felt first) and the test stimulus. Length, area, volume and curvature are all relevant (geometric) properties of the stimuli that might cause aftereffects. Therefore, we will first discuss such aftereffects before introducing the research questions in more detail.

Length was the first property that was studied systematically in this context. Köhler and Dinnerstein [3] showed an effect similar to that of the temperature aftereffect: to the hand adapted to a bar with a larger width than the test, the test felt relatively small, and vice versa. Cameron [4] performed a similar experiment with an additional condition where the hands were crossed. As crossing the hands did not change the results, they concluded that the aftereffect originated in the hand and not in phenomenal space. Using similar stimuli, Walker and Shea [5] concluded that this tactual size aftereffect is contingent on hand position as they failed to find any indication of intermanual transfer.

Uznadze [6] was one of the first to report on the aftereffect of three-dimensional size. In his experiment, participants had to repeatedly grasp two spheres of different sizes, one hand always grasping the larger one and the other the smaller. After 10 to 15 successive grasps, the hands were presented with test spheres of intermediate size. To the hand adapted to the larger sphere, the test sphere felt small, to the other hand it felt large. In a similar experiment, Maravita [7] was able to replicate this haptic size aftereffect. Kappers and Bergmann Tiest [8] measured this effect quantitatively by means of a size bisection task. They reported a difference in perceived size between the hand adapted to the large sphere and the hand adapted to the small sphere of 24% for adaptation spheres of 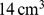 and 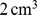, respectively.

As curvature is an obvious property of a sphere, it is also important to consider haptic curvature aftereffects, as the size aftereffect of the spheres might be either due to their size or their curvature. Gibson [9] reported that after following a curved line with a finger during 3 minutes, a subsequently stroked straight line felt curved in the opposite direction. Vogels and colleagues [10], [11] showed that for hand-sized three-dimensional surfaces, aftereffects are already measurable after an adaptation period as short as 2 s. The strength of this aftereffect saturated within 10 to 15 s, but it persisted at least 40 s. They also showed that the aftereffect could not be peripheral, as changing the kinesthetic input by bending and stretching the fingers or making a fist after adaptation but before testing, did not influence the strength of the aftereffect.

An additional point of interest here is the finding by Kahrimanovic et al. [12] that when participants are asked to compare sizes (i.e. volumes) of different shapes, they typically use surface area instead of volume to perform this task. However, in the haptic size aftereffect experiment of Kappers and Bergmann Tiest [8], participants did on average use volume. At this moment, it is still unclear whether this difference is due to the different tasks (volume discrimination versus size bisection after adaptation) or the (dis)similarity of the stimuli (a sphere and a tetrahedron versus just spheres).

The primary research question of the current study is whether, and if so how, stimulus shape influences the strength of the haptic size aftereffect. For this purpose, the stimuli will consist of spheres and tetrahedra. Important differences between such shapes are the curvature of the spheres and the edges of the tetrahedra. A tetrahedron is the simplest of all convex polyhedra and it has the smallest number of edges and faces possible. A sphere is the smoothest convex shape. If the aftereffect in the experiments with the spheres was mainly due to their curvature, it might be expected that after adaptation to spheres, the aftereffect is diminished or even vanishes when testing with tetrahedra as compared to testing with spheres. On the other hand, if size is the major contributor to the aftereffect, as could be expected on the basis of the experiments with the bars [3]–[5], the strength of the aftereffect might be independent of shape.

The second aim of this study is to investigate which geometric property (volume, area or length) participants use (either consciously or unconsciously) in the haptic size bisection task using either similarly or differently shaped stimuli. The results of Kappers and Bergmann Tiest [8] suggest that volume will be used, at least in the case of similar stimuli. However, on the basis of the results of Kahrimanovic et al. [12] one might expect the use of surface area when the shapes to compare are different.

In order to answer these research questions, four experimental conditions were tested. In all cases, the experimental task will be a size bisection task after adaptation to stimuli of different sizes, as used by Kappers and Bergmann Tiest [8]. Spheres and tetrahedra will be used both as adaptation and test stimuli, leading to four different combinations. This experimental design will provide answers to both research questions as will be explained in more detail in the Methods section.

## Methods

### Participants

16 students from VU University Amsterdam (9 females and 7 males) participated voluntarily in this experiment. 13 participants were right-handed and 3 were left-handed as assessed by means of a standard questionnaire [13]. Their mean age was 23 years and ranged from 19 to 27 years. None of these participants took part in the earlier experiment by Kappers and Bergmann Tiest [8]. Before the experiments started, they signed a declaration of informed consent. The experimental design was approved by the Ethical Committee (ECB) of the Faculty of Human Movement Sciences at VU University Amsterdam. All participants were unaware of the aims and background of the experiment. They received a monetary compensation of 12.50 Euro for their participation. Each participant took part in all 4 experimental conditions. One of the original participants was replaced as from observations by the experimenter and debriefing afterwards it became clear that in her first blocks the experimental task was not quite clear to her.

### Set-up and Stimuli

**Figure 1:**
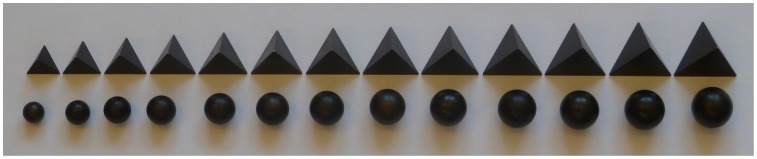
Stimuli used in the experiment. Top row: tetrahedra; bottom row: spheres. Both types of stimuli ranged in volume from 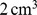 to 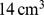 in steps of 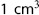. The two outer stimuli of each type were used as adaptation stimuli.

**Figure 2:**
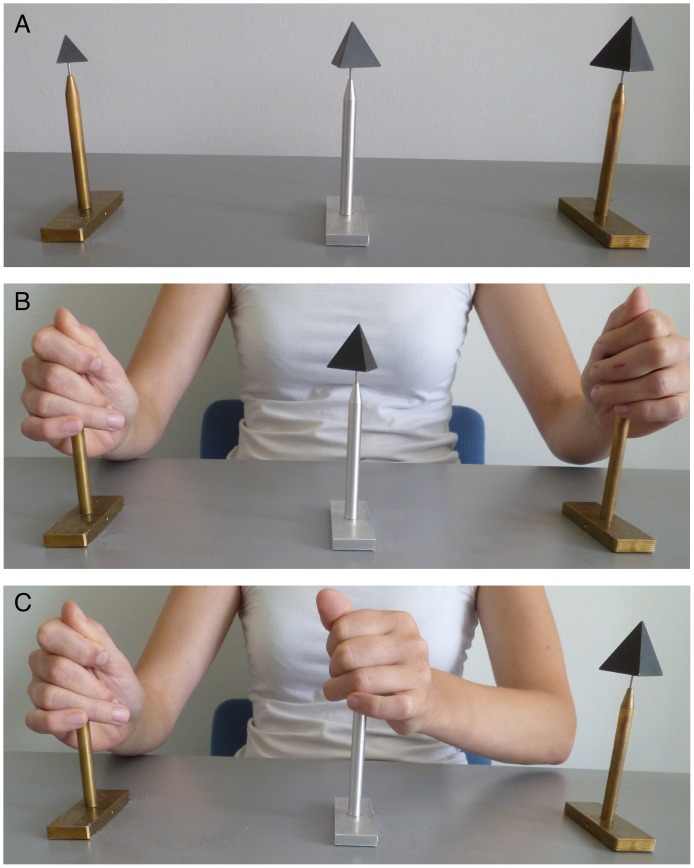
Experimental set-up. A) The stimuli, in this case the tetrahedra, are placed on small stands. The two outer stimuli always have volumes of 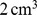 and 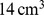; the volume of the middle (test) stimulus varies according to staircase procedures. B) The participant grasps the two outer stimuli for adaptation to their size. C) The participant grasps the test stimulus and decides whether the size of this test stimulus is larger or smaller than halfway between the two outer stimuli. As quickly as possible the participant returns to the adaptation configuration in B.

Stimuli were small brass spheres and tetrahedra with volumes of 2 to 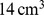 in steps of 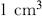 (see Figure 1). These same stimuli were used earlier in a volume discrimination experiment [12]. We have chosen tetrahedra in preference to cubes because in geometrical terms they are more different from spheres than cubes and because they caused larger effects than the cubes in the volume discrimination experiment. A cylindrical hole was made in the stimuli so that they could be placed on small stands (see Figure 2A). The distance between the two outer stands was 40 cm and these were placed symmetrically in front of the participant. Halfway between these stands a third stand was placed, 24.5 cm from the edge of the table. On this third stand the test stimulus could be placed.

### Experimental Conditions

There were 4 experimental conditions. In the SS (Sphere-Sphere) condition, spheres were used both as adaptation and as test stimuli. In ST (Sphere-Tetrahedron), the spheres were used as adaptation stimuli and the tetrahedra as test stimuli. In TT (Tetrahedron-Tetrahedron), the tetrahedrons were used both as adaptation and as test stimuli. Finally, in TS (Tetrahedron-Sphere), the tetrahedra were used for adaptation and the spheres for testing. Each experimental condition consisted of 4 blocks (explained below) which were finished before measuring another condition. Measuring all four conditions took somewhat less than 1.5 hour, including a short (coffee) break halfway.

### Procedure

At the start of the experiment, the participants were asked to blindfold themselves. Next, the experimenter guided their hands to the two outer stimuli, depending on the experimental condition, the spheres or the tetrahedrons of 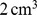 and 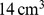. The order of the experimental conditions, and thus also the placement (right/left) of the small and large adaptation stimuli, was counterbalanced over participants. Participants were asked to grasp the stimuli as shown in Figure 2B in order to get a good impression of their sizes. This was the start of the first adaptation phase, which lasted at least 1.5 min. During this adaptation phase, the experimenter explained the size bisection task.

After the adaptation phase, the test phase started. Within a block of 20 trials, participants had to use one predetermined hand. With this hand they had to grasp the test stimulus and decide as quickly as possible whether the size of this stimulus was smaller or larger than halfway between the two outer stimuli. They were supposed to immediately return to the adaptation stimulus. The hand that was not used for testing during a block, remained at the adaptation stimulus continuously, as shown in Figure 2C. Participants did not receive any feedback about the correctness of their answer. The experimenter fed the participant’s answer into a computer program, which computed the size of the next test stimulus (see below for more details). As the participants touched the test stimuli only briefly (typically less than a second), a top-up adaptation of a few seconds between trials was deemed to be sufficient to keep up the level of adaptation.

A block of 20 stimuli with one hand was followed immediately by a block of the same experimental condition and placement of the adaptation volumes with the other hand as test hand. In this way, a new adaptation phase between these blocks could be avoided. After two such blocks, the two outer stimuli were interchanged and a new adaptation phase of at least 1.5 min was necessary. This phase was either used for filling in the handedness questionnaire or filled with some small talk. Thus, there were 4 blocks for each experimental condition, that could be presented, for example, in the following order: adaptation phase; block 1: test hand right, adaptation volume 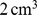 (R2); block 2: test hand left, adaptation volume 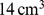 (L14); adaptation phase; block 3: test hand right, adaptation volume 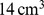 (R14); block 4: test hand left, adaptation volume 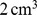 (L2).

**Figure 3:**
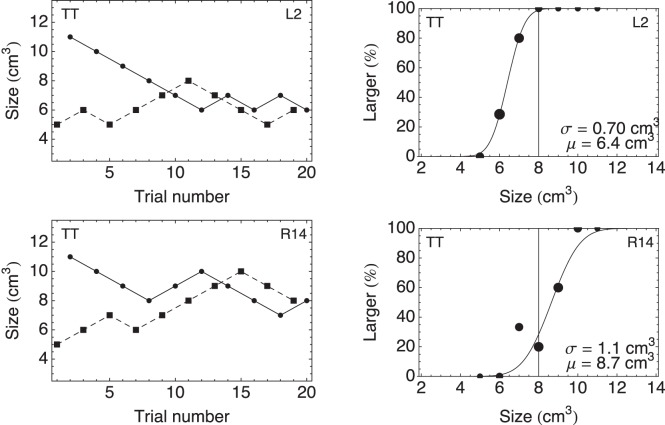
Staircases and psychometric curves. Examples of the staircases (left) and the resulting psychometric curves fitted to the data points (right) for one participant in the condition TT. One of the staircases in a panel started with a volume of 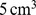 (dashed lines, squares) and the other with 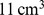 (solid line, disks). In the upper panels, the participant’s left hand, adapted to a size of 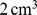, was used as test hand (L2). In the lower panels, the right hand, adapted to a size of 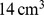, was used as test hand (R14). The difference between the 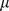
s gives an indication of the size of the aftereffect for this condition.

Within in a block, the volume of the test stimulus was determined by means of two interleaved one-up-one-down staircases. The first of these staircases started with a volume of 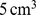 and the second with 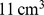 (see Figure 3, left panels). After an answer “larger”, the subsequent volume of the test stimulus in that staircase was made one step (i.e. 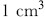) smaller, and vice versa. The possible test volumes ranged from 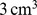 to 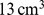. In a total of 20 trials (10 for each staircase), the volume that was perceived as halfway between the outer two volumes was determined.

### Order of the Conditions

The order of the experimental conditions was counterbalanced over participants, taking into account the following rules: 1) The 4 blocks belonging to an experimental condition were finished before measuring another condition. 2) Participants either started with the two conditions in which spheres were used for adapation, or with those two in which the tetrahedra were used for adaptation. 3) Blocks using the right hand were always interspersed with blocks using the left hand. 4) The placement of the volumes in the first block of a new experimental condition was always the same as in the last block of the previous condition (to make optimal use of the existing adaptation).

Thus, half of the participants started with the spheres as adaptation shapes, the others with the tetrahedra. Half of the participants started with their right hand as test hand, the others with their left hand. Half of the participants started with the larger volume at their right, the others with the smaller volume. Taken all these rules together, there are 16 possible orderings, which explains our choice of 16 participants.

### Data Analysis

For each participant and each condition the percentage of “larger” answers was plotted as a function of volume (see Figure 3, right panels). As we were interested in the Point of Subjective Equality (PSE or the 50% point) of the psychometric curve, we fitted the following cumulative Gaussian function
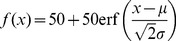
(1)to the data. Here, 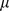 is the volume that a participant perceives as lying halfway between the two outer volumes of 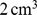 and 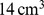 (i.e. the participant guesses in 50% of the trials that the volume is larger and in the other 50% that it is smaller); 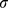 is a measure of the steepness of the curve and is the difference between the 50% and the 84% point; 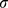 is also termed threshold. In this study we will use the values of 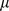 to compare the various conditions.

The aftereffect in an experimental condition is determined from the data of all four blocks belonging to that condition, and is defined as follows:
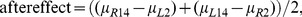
(2)where the subscripts indicate the specific block. Basically, this is the average of the aftereffects of the two different placements of the stimuli.

### Volume, Surface Area and Linear Dimensions

The average value of 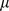 over the four blocks of an experimental condition gives an indication of which geometrical parameter participants used for the determination of the halfway volume. In this experiment, a volume of 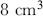 always lies exactly halfway the two adaptation volumes. However, in the study of Kahrimanovic et al. [12] it was found that when participants have to compare the volumes of different shapes, they use surface area as a measure of size. When surface area was removed, they tended to use a linear measure of size. As volume, area and length are related in a non-linear way, different measures will lead to different perceived halfway volumes. Table 1 shows the different halfway volumes if participants based their decision on different geometrical measures. It can be seen, for example, that if participants used the radius (diameter) as a measure of size in the SS condition, the expected halfway volume (the average of the 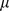
s over the four blocks) would be 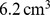.

**Table 1:** Halfway volumes based on different geometrical properties.

reference	shape	*dl* (cm)	*A* (cm^3^)	*V* (cm^3^)	halfway *V* (cm^3^)
sphere	small reference sphere	1.56	7.7	2.0	
	large reference sphere	2.99	28.1	14.0	
	halfway sphere w.r.t. volume			**8.0**	8.0
	halfway sphere w.r.t. area		**17.9**		7.1
	halfway sphere w.r.t. radius	**2.28**			6.2
	halfway tetrahedron w.r.t. volume			**8.0**	8.0
	halfway tetrahedron w.r.t. area		**17.9**		3.9
	halfway tetrahedron w.r.t. radius	**2.28**			1.4
tetrahedron	small reference tetrahedron	2.57	11.4	2.0	
	large reference tetrahedron	4.92	41.9	14.0	
	halfway sphere w.r.t. volume			**8.0**	8.0
	halfway sphere w.r.t. area		**26.6**		12.9
	halfway sphere w.r.t. radius	**3.74**			27.5
	halfway tetrahedron w.r.t. volume			**8.0**	8.0
	halfway tetrahedron w.r.t. area		**26.6**		7.1
	halfway tetrahedron w.r.t. radius	**3.74**			6.2

Relationships between volumes (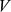), surface areas (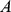), radii (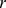), diameters (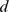) and edge lengths (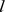) of spheres and tetrahedra. The following formulas hold for the sphere: 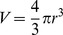, 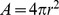 and 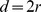, and for the tetrahedron: 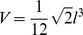 and 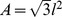. Adaptation volumes are always 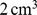 and 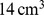 and thus the correct halfway volume is 8 cm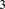. The values in bold are the averages of the corresponding values of the two reference stimuli. The rightmost column gives the resulting halfway volumes if participants based their judgements on the indicated geometric property.

### Statistical Analysis

As the primary aim of this study is to investigate whether the various conditions lead to aftereffects, we will first present the results for the individual conditions. We do not consider an overall ANOVA appropriate, as only comparisons between the aftereffects obtained in SS and TT, in SS and ST, and in TT and TS are relevant. For these comparisons, paired *t*-tests will be used, with a Bonferroni correction for multiple comparisons (3 in this case). For the mean volumes, the only relevant comparison is that between conditions ST and TS, so no correction is needed. In case the distribution of one of the data sets is not normal, we will use the Mann-Whitney test. We will use a significance level of 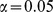.

## Results

### Aftereffects

#### Sphere-Sphere condition (SS)

**Figure 4:**
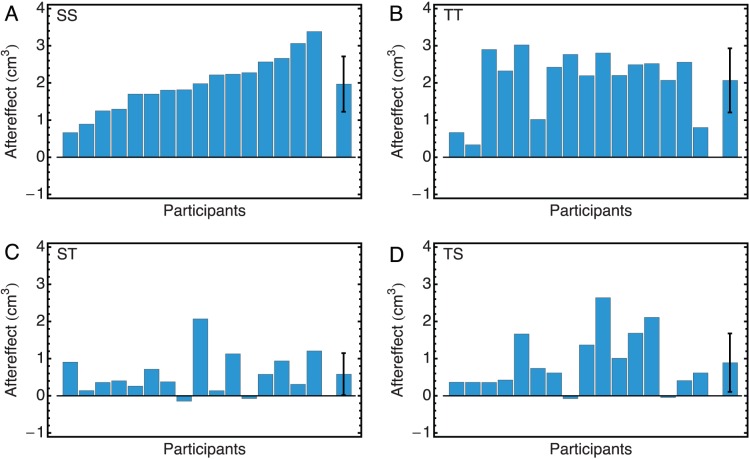
Aftereffects. Aftereffects for all 16 participants in all four conditions: A) SS; B) TT; C) ST; D) TS. A positive aftereffect indicates that after adaptation to a large volume, subsequently touched stimuli were perceived as smaller than after adaptation to a small volume. The rightmost bar shows the average over all participants with the standard deviation. The participants are sorted according to the size of their aftereffect in the SS condition.

The aftereffects obtained by the individual participants in the SS condition are shown in Figure 4A. The order of the participants is according to the size of their aftereffect and this order is kept the same in all other panels and figures. It can be seen that all aftereffects were positive, indicating that after adapting to the larger sphere, the test sphere was perceived as relatively smaller than after adaptation to the smaller sphere. The mean aftereffect over participants was 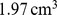, which is 25% (aftereffect as percentage of the test volume of 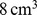). This aftereffect was highly significant as determined by a *t*-test (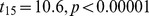).

The steepness of the psychometric curves, as measured by 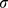, gives an indication of the reliability of the participants. Moreover, a 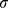 smaller than the aftereffect gives extra support for the existence of the aftereffect. In the SS condition, the mean 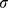 was 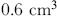. This was indeed much smaller than the aftereffect (one-sided paired *t*-test: 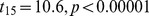).

#### Tetrahedron-Tetrahedron condition (TT)

The aftereffects in the TT condition are shown in Figure 4B. The average value of the aftereffect was 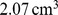. It can be seen that there are quite some individual variations. Again, the aftereffect was significantly different from 0 (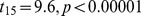). Also in this condition, the mean 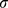 of 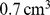 was significantly smaller than the aftereffect (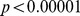).

#### Sphere-Tetrahedron condition (ST)

The aftereffects in the ST condition are shown in Figure 4C. In this condition, many of the aftereffects were quite small and some were even negative. The mean aftereffect was 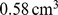, which was still significantly different from 0 (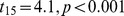). However, the aftereffect was not significantly different from 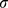 (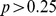).

#### Tetrahedron-Sphere condition (TS)

The aftereffects in the TS condition are shown in Figure 4D. Again there was quite some variation over the participants with both relatively large and negative values being present. The mean value of the aftereffect was 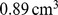, which was significantly different from 0 (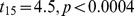). Like in the ST condition, the aftereffect was not significantly different from 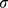 (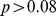).

#### Comparisons between conditions

The aftereffects in the conditions in which the same shapes were used (SS and TT) were larger than those in the conditions where the adaptation and test stimuli differed in shape (ST and TS): For 14 out 16 participants the ST aftereffect was smaller than the SS aftereffect and for 15 out of 16 participants the TS aftereffect was smaller than the TT aftereffect. This difference was highly significant (SS versus ST: paired *t*-test gave 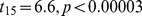, and TT versus TS: Mann-Whitney test gave 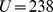, 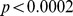 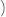 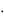 There was no significant difference between the aftereffects of the conditions SS and TT (Mann-Whitney test: 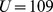 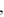 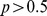).

Although these significant differences might suggest that the aftereffects in these two conditions were similar, it can be seen in Figures 4A and B, that there was no systematic pattern over participants. Thus, it was not the case that participants with a small/large aftereffect in SS had also a relatively small/large aftereffect in TT. Indeed, the correlation between the aftereffects obtained in these two conditions, 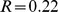, was far from significant (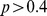).

### Mean Volumes

#### Sphere-Sphere condition (SS)

**Figure 5:**
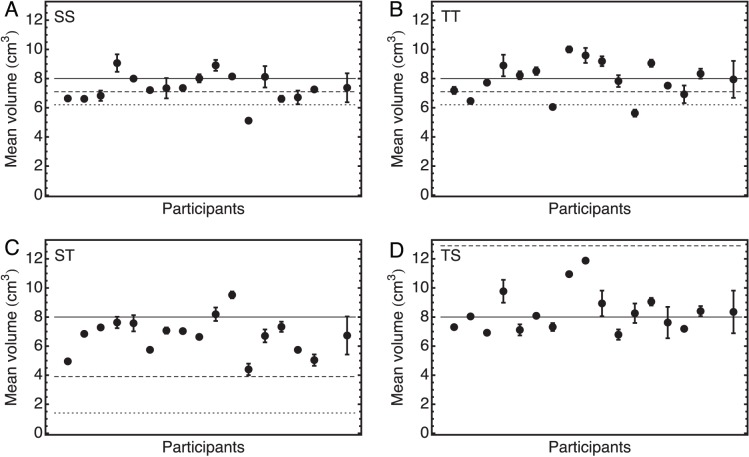
Mean halfway volumes. Volume that was perceived as halfway between the adaptation stimuli with volumes 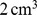 and 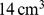 for all participants in all four experimental conditions: A) SS; B) TT; C) ST; D) TS. The veridical halfway volume is 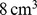, as indicated with the solid line. The dashed lines indicate the expected mean volume if participants used area (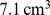 for SS and TT, 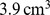 for ST and 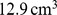 for TS). The dotted lines indicate the expected mean volumes if participants used a linear dimension (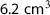 for SS and TT, and 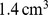 for ST; the value of 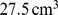 for TS falls outside the plotted range). The error bars indicate the range of the mean volumes obtained in the two different placements of the stimuli. The rightmost data point in each graph shows the mean over all participants with the standard deviation. The participants are ordered in the same way as in Figure 4.

The mean volume in the SS condition can be seen in Figure 5A. The mean volume over all participants was 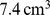. This was significantly smaller than the veridical value of 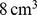 (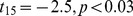). This mean volume was not significantly different from 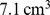, the value to be expected if participants used area to judge size (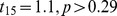). It can also be seen that participants varied widely in their mean volume, which ranged from 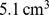 to 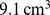. This range was as large as 50% of the reference volume. However, the participants themselves were quite consistent as indicated by the relatively small error bars, that sometimes are not even visible. These error bars indicate the range of the means of a participant obtained in the two different placements of the reference spheres.

#### Tetrahedron-Tetrahedron condition (TT)

The mean volumes in the TT condition are shown in Figure 5B. The average value over all participants was 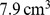, which was not significantly different from 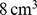 (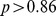). However, it can also be seen that there are quite substantial individual differences, with the mean volume ranging from 5.6 to 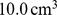.

#### Sphere-Tetrahedron condition (ST)

In Figure 5C, the mean volumes obtained in the ST condition are shown. The mean volume averaged over all participants was 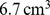. This was significantly smaller than 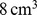 (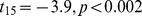), but all means were substantially above 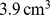. Once again, the means of the individual participants varied widely, from 4.4 to 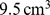, but the participants themselves were quite consistent, as can be concluded from the relatively small error bars.

#### Tetrahedron-Sphere condition (TS)

Finally, the mean volumes in the TS condition are shown in Figure 5D. The mean volume over all participants was 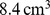, which did not differ significantly from 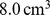. All values were substantially smaller than 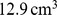. Also in this condition, the participants varied widely in their mean volume, namely from 6.8 to 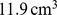.

#### Comparisons between conditions

The only relevant comparison of mean volumes between conditions is that between ST and TS. If participants did not use volume as a measure for size but another geometric property such as surface area or length, this would lead to mean volume changes in opposite directions for these two conditions. The significant difference (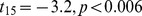) between the obtained means (ST 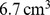, TS 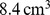) indicates that this was indeed the case. There was, however, no significant correlation (or anti-correlation) between the mean volumes obtained in these conditions (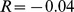, 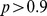).

## Discussion and Conclusions

The first research question could be answered unequivocally: haptic size aftereffects clearly depended on shape and, more specifically, on the combination of shapes used. As long as adaptation stimuli and test stimuli had the same shape, either spherical or tetrahedrical, a relatively strong aftereffect was obtained of about 25% of the reference volume of 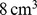. In conditions where adaptation and test stimuli were different in shape, the aftereffects were significantly smaller. The results in the Sphere-Sphere condition replicated those obtained in an earlier study [8], the other conditions were new.

These results raise the question what caused the aftereffect. The finding that both spheres and tetrahedra could produce substantial aftereffects suggests that size is the major factor and not curvature or sharpness of edges. However, this is contradicted by the finding that shapes needed to be the same in order to induce large aftereffects. Moreover, if size were the major factor, one would expect a high correlation between the aftereffects obtained in the Sphere-Sphere and the Tetrahedron-Tetrahedron conditions, but such was not the case. These findings lead to the conclusion that these aftereffects are not actually effects of size alone but of the combination of shape and size. We expect this finding to be true in general and not specifically for the combination of spheres and tetrahedrons. Probably, the more similar adaptation and test shapes, the stronger this after-effect.

That shape can indeed lead to haptic aftereffects was shown recently by Matsumiya [14] who reported a haptic face aftereffect. During 20 s participants haptically adapted with their eyes closed to either a face with a happy expression or one with a sad expression. The subsequently explored neutral test face was judged more often as happy after adaptation to a sad face than after adaptation to a happy face, and vice versa. Faces and especially facial expressions are very specific instances of shapes and processing occurs at corticial areas such as inferior frontal gyrus, inferior parietal lobe, and superior temporal sulcus [15]. Therefore, Matsumiya [14] hypothesized that the haptic face aftereffect may be associated with adaptation in these higher cortical areas. Although Kitada et al. [15] showed that haptic identification of faces involves different areas than in the case of non-facial objects like shoes, it still seems likely that the haptic shape-size aftereffects also originate at higher cortical areas and do not have a peripheral origin. This is further supported by the findings that haptic curvature aftereffects do not diminish after peripheral stimulation [11] and that, in some conditions, these aftereffects are transferred to the other hand [16], [17].

The answer to our second research question, determining the geometric property that participants use in the haptic size bisection task, remains inconclusive. As can be seen in all panels of Figure 5, participants were very consistent over the two different placements of the stimuli (small, sometimes negligible error bars), but they varied widely in the geometric property they seemed to use. In the case of adaptation to spheres, the mean volume over all participants was significantly smaller than 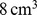, suggesting that participants used surface area. In our previous, smaller, study in which only the Sphere-Sphere condition was measured [8], the mean volume did not differ from 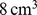, but if all participants from both studies are taken together, the difference remains significant (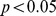). The use of surface area would be consistent with results by Kahrimanovic et al. [12], who reported that in the discrimination of volumes of different shapes such as spheres, cubes and tetrahedra, participants used surface area, although they were explicitly instructed to use volume. However, in our experimental conditions where the adaptation shapes were tetrahedra, the mean volume did not differ from 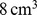, not even when the test shapes were spheres. This was clearly not consistent with the findings of Kahrimanovic et al. [12].

As in all conditions the participants varied so widely, the only conclusion we can draw is that in this haptic bisection task, participants used different strategies but in a consistent way. This also means that in most of the conditions participants did not use a simple geometric property such as area or volume, as can most clearly be inferred from panel C in Figure 5: the error bars were very small, but almost none of the participants were clearly using area or volume. The question which geometric property participants did use is hard to answer from our data. They might have used some combination of cues, possibly depending on the specific task (discrimination or bisection), but clearly more research is needed.

In summary, the main finding of this study is that haptic size aftereffects strongly depend on shape, and size alone could not be the determining factor. Therefore, it would be more appropriate to term these aftereffects haptic shape-size aftereffects.
